# Mapping the immunological landscape and emerging immunotherapeutic strategies in cervical cancer: a comprehensive review

**DOI:** 10.3389/fonc.2025.1620501

**Published:** 2025-07-10

**Authors:** Xinyi Zhang, Wenyang Nie, Wenwen Shao, Qian Guo

**Affiliations:** ^1^ Clinical Medical College, Southwest Medical University, Luzhou, China; ^2^ College of First Clinical Medicine, Shandong University of Traditional Chinese Medicine, Jinan, China; ^3^ Department of Gynecology, Affiliated Hospital of Southwest Medical University, Luzhou, China

**Keywords:** cervical cancer, tumor immune microenvironment, immunotherapy, PD-1/PD-L1 signaling pathway, immune checkpoint

## Abstract

Cervical cancer continues to pose a considerable global health challenge, especially in low- and middle-income nations, although progress in screening and vaccine efforts. In recent years, immunotherapy has emerged as a promising treatment option; nevertheless, its efficacy in cervical cancer is constrained by the intricate and heterogeneous tumor immune microenvironment. Reliable biomarkers to predict which patients will benefit from immunotherapy are lacking. The heterogeneity of the immune landscape across patients adds further complexity. This paper offers a thorough examination of the immunological landscape in cervical cancer, highlighting the interactions among tumor cells, immune infiltrates, and stromal elements. Moreover, we investigate how advanced technologies—such as single-cell RNA sequencing, spatial transcriptomics, and multiplex imaging—are transforming our comprehension of immunological heterogeneity and uncovering new therapeutic targets. We seek to delineate present problems and potential pathways in the development of effective, tailored immunotherapies for cervical cancer by integrating genetic analysis with immunological insights.

## 1 Introduction

Cervical cancer (CC) ranks as the fourth most common malignant neoplasm among women globally, serving as a primary contributor to cancer mortality in females (1, 2). It is a serious threat to women’s health. CC is preventable and treatable, but remains a major global health burden (3). Alarmingly, more than 85% of these cases and deaths occur in low- and middle-income countries, where access to HPV vaccination, routine screening, and timely treatment is often limited. CC can be categorized into squamous cell carcinoma (SCC), adenocarcinoma (AC), and adenosquamous carcinoma (ASC), which represent the three predominant histological variants of CC (4–6). Human papillomavirus (HPV) is a requisite yet insufficient factor in the etiology of CC. HPV is a prevalent sexually transmitted infection; nevertheless, the majority of HPV infections are eradicated by the immune system. Persistent infection of the genital mucous membranes by specific high-risk HPV types (HPV16 and HPV18) induces cellular proliferation and genetic instability, which, if unaddressed, may ultimately progress to malignant tumors (7–10).

In recent years, CC incidence and mortality have declined significantly in some developed countries due to advances in early screening methods and increased HPV vaccine coverage, but CC cannot be completely prevented by this strategy alone (11–16). Presently, commercially accessible preventive HPV vaccinations are prevalent; nevertheless, they do not manage confirmed infections or lesions (17–20). First-line treatment options for CC are still limited to traditional methods such as surgical excision, radiotherapy and chemotherapy (21–25). Treatment alternatives for early and locally invasive CC encompass radical hysterectomy or radical hysterectomy in conjunction with pelvic lymph node dissection, and concomitant chemotherapy and radiotherapy (26–29). Treatment for distant metastatic CC emphasizes systemic therapy. There is no standard treatment for second-line systemic therapy for advanced CC (30, 31). Targeted agents such as tisotumab vedotin and bevacizumab can help some advanced metastatic patients who meet the criteria (32–34), but as the disease worsens or resistance develops, options for further treatment are few and the toxicity and decreased quality of life they cause cannot be ignored. These factors make treating advanced and repeated metastatic CC a very tough task in clinical practice. How to find more accurate biomarkers to help with personalized and accurate treatment has become a major scientific problem that needs to be solved in the current field of CC study.

A key advance in addressing the therapeutic challenges associated with advanced and metastatic CC has been the advent of immunotherapy (35, 36). Immunotherapy is considered a groundbreaking modality in contemporary oncology and offers promising avenues for tumor control (37–42). Immunotherapy, as a novel treatment approach, aims to augment the body’s innate and adaptive immune responses to combat cancer cells. This approach encompasses a range of modalities, including immune checkpoint inhibitors (ICIs), monoclonal antibodies, cancer vaccines, immunomodulatory agents, and adoptive T-cell transfer therapies (43–45). Each of these strategies aims to overcome mechanisms of immune evasion and restore effective antitumor immunity, offering promising avenues for improving outcomes in various malignancies (46, 47). The use of ICIs has brought new hope to patients (48, 49). In clinical practice, inhibitors targeting the immune checkpoints PD-1 and CTLA4 have been shown to improve survival in patients (21, 50–53).

However, the unique histologic features of CC pose a significant challenge to the heterogeneity of immunotherapy (54, 55). Clinical data suggest that only 15-20% of patients benefit from ICIs therapy, and reliable predictors of efficacy are lacking (56). This event really shows how complicated and varied the CC tumor microenvironment (TME) regulatory network is. This means we need to learn more about how it works on the inside and look into how different kinds of immune cells get into the TME in order to make immunotherapies work better and come up with new ways to treat cancer. Emerging tools such as spatial transcriptomics, multiplex imaging, and single-cell RNA sequencing allow high-resolution mapping of the TME (57–62). These approaches are expected to uncover novel immunoregulatory pathways, improve biomarker discovery, and support the development of highly personalized immunotherapy regimens (63–66). Combining multi-omics techniques has become a useful way to study the complexity of TME in recent years (67–70). By correctly looking at the molecular features of each cell group in TME, the dynamic interaction network between tumor cells and immune cells is made clear.

**Figure 1:**
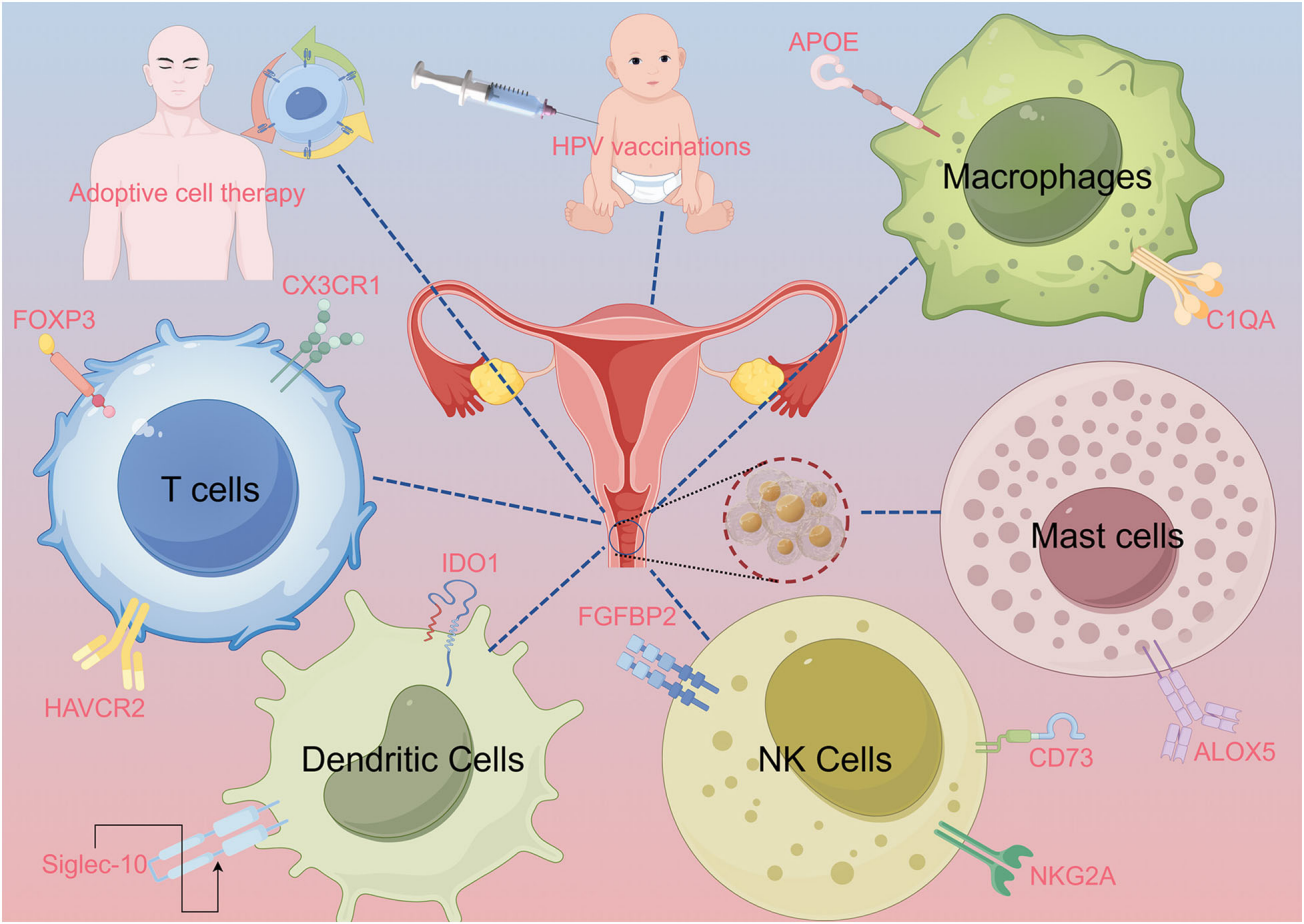
Abstract image. The abstract figure illustrates various scenarios of interactions between immune cells and cervical cancer. These include T cells, dendritic cells (DCs), natural killer (NK) cells, macrophages, and mast cells, with relevant proteins and signaling pathways labeled. The image of a syringe and an infant symbolizes human papillomavirus (HPV) vaccination. Adaptive cell therapy is explained using a human figure. Lines connect the various elements to the central image of the cervix, highlighting their immune interactions.

This review aims to systematically examine the immunological landscape of CC, focusing on how tumor–immune interactions contribute to therapeutic resistance and immune evasion. We place particular emphasis on novel immune targets identified through single-cell and spatial transcriptomic technologies, and we explore how these insights may inform next-generation immunotherapeutic strategies (**Figure 1**).

## 2 Variability of immune responses in TME of CC

Even though there have been big improvements in CC therapy with ICIs over the last ten years, a lot of patients are still not getting the best immune responses in clinical situations. The heterogeneity of therapeutic response may be influenced by the characteristics of immune cell infiltration in the tumor microenvironment, the integration status of the HPV genome, the expression profiles of immune-related biomarkers, and individual differences in host immune status (21, 71–73). Persistent infection with high-risk HPV, particularly HPV16 and HPV18, plays a central role in cervical carcinogenesis. These viruses have evolved several sophisticated strategies to evade host immune surveillance, which contributes to both viral persistence and progression to malignancy. The HPV oncoproteins E6 and E7 disrupt multiple immune pathways (74). E6 targets interferon regulatory factors (IRFs) and hinders Type I interferon responses, limiting antiviral immunity (75). E7 interferes with antigen processing and presentation, reducing cytotoxic T lymphocyte (CTL) recognition of infected cells (76). Both E6 and E7 reduce the function of dendritic cells (DCs) and Langerhans cells, impairing the priming of HPV-specific T cells (77). The resulting TME becomes immunosuppressive, posing significant barriers to the success of immunotherapies. Cervical tumors, like many solid tumors, create a hostile microenvironment for effective immune activation. PD-L1 is commonly expressed in cervical tumors, especially in HPV-positive cases, leading to T cell exhaustion via the PD-1/PD-L1 axis. Understanding these evasion strategies is critical to improving the response to immune checkpoint inhibitors and other immunotherapies in CC.

The TME displays dynamic and intricate characteristics across different cancer types, where immune cells play a crucial role in the elimination of tumor cells; but, in some cases, they may also promote tumor progression (78–80). In hypermetabolic tumor regions, there were stronger signals from CD56 Natural Killer (NK) cells and immature dendritic cells, while hypometabolic tumor regions exhibited a higher presence of eosinophils, immature B cells, and Treg cells (81). Fan et al. performed a multi-omics investigation demonstrating that bidirectional interactions between malignant epithelial cytokeratin cells and immune-suppressive cancer-associated fibroblasts foster an immune rejection microenvironment in CC through FABP5-mediated transforming growth factor β pathway signalling (82). Additionally, specific molecular processes indicate that NAT10-mediated metabolic reprogramming in cancer cells inhibits the therapeutic efficacy of PD-L1 blockade therapy (83). Insights into the complex immunological landscape of the TME have paved the way for novel treatment strategies (84–88). Finding new immunotherapeutic targets and quickly putting possible biomarkers into clinical trials are big steps forward in improving clinical response rates, making combination therapy strategies work better, and dealing with immunotherapeutic heterogeneity. The following sections will explore current immunotherapeutic approaches and emerging targets that aim to overcome the barriers posed by immune heterogeneity in CC.

## 3 Summary of contemporary immunotherapy approaches

### 3.1 Immune checkpoint inhibitors

Immune checkpoints are molecules that inhibit signalling pathways and uphold immune tolerance; however, cancer cells frequently exploit these mechanisms to escape immune surveillance (89, 90). ICIs represent a significant advancement in tumor therapy. Their mode of action primarily involves augmenting the cytotoxic effect of T lymphocytes through the targeted inhibition of inhibitory receptors. ICIs targeting the PD-1/PD-L1 signalling pathway, such as Pembrolizumab, Nivolumab, Cemiplimab, and Balstilimab, have received approval for second-line and subsequent treatment of advanced CC (91–94). While immunotherapy offers a promising avenue for improving outcomes in cervical cancer, it is not without risks. The use of ICIs can lead to a spectrum of immune-related adverse events resulting from excessive immune activation (95). As such, careful patient selection, early detection of toxicity, and proactive management protocols are essential components of clinical immunotherapy use. The therapeutic application of ICIs has progressively transitioned from monotherapy to combination therapy techniques. Research has demonstrated that the dual ICIs regimen exhibits substantial efficacy in patients with metastatic or recurrent CC (96). The combination of ICIs with anti-angiogenic agents, such as Sintilimab and Anlotinib, has demonstrated significant therapeutic efficacy in patients with advanced CC who have not responded to chemotherapy. In patients with advanced CC who have not responded to first-line platinum-based therapy, the overall response rate (ORR) of Sintilimab in conjunction with albumin-paclitaxel is 44.4%, with a median progression-free survival (mPFS) of 5.2 months and a median overall survival (mOS) of 13.1 months (97). The treatment strategy of ICIs combined with concurrent chemoradiotherapy has demonstrated considerable success in patients with high-risk locally advanced CC. Combining radiotherapy and chemotherapy with immunotherapy holds significant promise for improving treatment outcomes in CC. Radiotherapy can enhance tumor immunogenicity by inducing immunogenic cell death, increasing antigen presentation, and modulating the tumor microenvironment to promote immune infiltration. When paired with ICIs, these effects may synergize to overcome immune resistance. Ongoing clinical trials are exploring triplet combinations of radiotherapy, chemotherapy, and PD-1/PD-L1 inhibitors (98). Looking forward, integrating immunotherapy with standard treatments may enable personalized therapeutic strategies.

### 3.2 Therapeutic vaccinations for HPV

Through the activation of certain T cell immunological responses, the therapeutic vaccine method that is based on HPV oncoprotein E6/E7 attempts to generate anti-tumor effects. A phase II clinical trial (NCT04405349) validated the long-term clinical benefit of the therapeutic DNA vaccine VB10.16 combined with atezolizumab in the population of HPV16-positive patients with recurrent or metastatic CC. The trial demonstrated an overall response rate (ORR) of 19.1%, a median progression-free survival (mPFS) of 4.1 months, and a median overall survival (mOS) that was prolonged to 21.3 months (99). Another phase II clinical trial (NCT04096911) indicated that the combination of Sintilimab and the HPV quadrivalent vaccine could markedly enhance clinical outcomes for individuals with recurrent or metastatic CC who were unresponsive or intolerant to traditional therapies. The treatment regimen had a median progression-free survival (mPFS) of 7.16 months, an overall response rate (ORR) of 53.8%, and a disease control rate of 76.9% on average (100). These research findings emphasize the necessity of spreading HPV preventive vaccination, especially in low-income areas with insufficient medical resources. Although therapeutic vaccines targeting E6/E7 have shown promise in early trials, their integration into standard care is hampered by low immunogenicity and logistical barriers in large-scale application. More rigorous phase III trials are needed to validate their long-term clinical benefit.

### 3.3 Adoptive cell therapy and application

ACT entails the ex vivo expansion of either autologous or allogeneic tumor-specific T cells, which are then reinfused into the patient to specifically target and eradicate tumor cells (101, 102). ACT is categorized into three primary types: tumor-infiltrating lymphocytes (TIL), TCR-engineered T cells (TCR-T), and chimeric antigen receptor T cells (CAR-T) (103–105). TILs demonstrated superior tumor abrogation compared to lymphocytes produced by vaccine treatment, indicating their efficacy in counteracting immune evasion mechanisms (106). Of nine patients with metastatic CC, two achieved complete remission and one had partial remission according to clinical investigations following TILs (107). Nisha et al. conducted the inaugural human phase 1 clinical study with TCR- T lymphocytes targeting E7 for metastatic human oncovirus-associated epithelial carcinoma, yielding significant tumor reduction and objective clinical responses in 6 out of 12 patients (108). In clinical trials, an NKG2D CAR-T treatment targeted for NKG2DL demonstrated great potential to drastically stop tumor development without appreciable off-target damage (109). These researches show a viable therapeutic method and emphasize the clinical possibilities of using cell therapy for the treatment of CC.

## 4 Potential novel therapeutic targets for CC in relation to immune infiltration

The extensive application of omics technologies, including scRNA-seq, has enabled researchers to achieve a single-cell comprehension of the TME. Immune infiltration, a significant element of the TME, has been demonstrated to influence tumor progression and the efficacy of immunotherapy. The expansion of options for CC immunotherapies, coupled with the significant functional heterogeneity and plasticity of immune cells, offers novel research avenues for creating personalized diagnostic and therapeutic strategies, as well as enhancing combination therapies.

### 4.1 T cells

The investigation of T cell-related immune checkpoints, as essential effector cells in tumor immunotherapy, has consistently been a primary focus for biomarker identification. The markers primarily regulate T cell activation, differentiation, and exhaustion.

HAVCR2, a significant member of the TIM family, demonstrates distinct expression characteristics in CC. Research indicates that HAVCR2 expression in exhausted CD8+ T cells within CC tissues is significantly elevated compared to precancerous lesions; however, this expression level declines following chemoradiotherapy (110). The expression level of the ligand-receptor pair LGALS9-HAVCR2 in the TME is significantly elevated in CC tissues compared to high-grade squamous intraepithelial lesions, potentially indicating enhanced immunosuppression (111). Exhausted CD8+ T cells in cervical adenocarcinoma exhibit high expression of HAVCR2 and TIGIT, whereas CD96 is predominantly expressed in exhausted CD8+ T cells in cervical squamous cell carcinoma. These molecules may serve as potential targets for immunotherapy (112, 111).

FOXP3 serves as a critical transcription factor for regulatory T cells (Tregs), with its expression level closely associated with the malignancy of CC. The elevated expression of FOXP3 correlates positively with the advancement of FIGO stage and the differentiation degree of histological subtypes in CC, and is significantly associated with a decreased overall survival rate in patients (113). The elevated expression of FOXP3 in HPV-related CC underscores its significant role in the immune evasion associated with this condition (114).

CX3CR1, a specific receptor for CX3CL1, plays a role in regulating the chemotaxis, adhesion, and cytotoxic functions of immune cells, in conjunction with the perforin-encoding gene PRF1. These two molecules are upregulated in effector memory T cells and cytotoxic T cells and serve a role in early immune activation within CC metastatic lymph nodes (115). The identification of the specific enrichment of CXCL13 in resident memory T cells, along with its immunosuppressive function, offers a novel perspective for a more comprehensive understanding of the complexities within the TME of CC (116).

### 4.2 Macrophages

The groundbreaking utilization of scRNA-seq technology has fundamentally transformed the conventional binary classification framework of macrophages. Researchers have progressed beyond categorizing macrophages solely as pro-inflammatory (M1 type) or anti-inflammatory (M2 type) (117), now delineating more nuanced functional subtypes based on their multifunctional attributes inside the TME.

APOE, a multifunctional protein released by hepatocytes and macrophages, has been recognized as a signature gene of lipid-associated macrophages due to its crucial role in lipoprotein clearance, lipid transport, and cholesterol metabolism. Macrophage subtypes that overexpress APOE can markedly augment the proliferative activity and migratory capacity of CC cells (118). Mechanistic investigations have demonstrated that this macrophage subtype may secrete immunosuppressive substances via exocytosis (110).

Li et al.’s work demonstrated the probable involvement of C1QA in the spread of CC. In comparison to primary CC tissues, macrophages exhibiting elevated C1QA expression were markedly enriched in metastatic lymph nodes, indicating that C1QA may play a role in the regulation of CC metastasis (119). A separate study arrived at a more intricate conclusion: in patients with advanced CC, macrophages exhibiting reduced SPP1 expression and elevated C1QA expression correlated with improved clinical outcomes (120). CD74 is a crucial regulator of the transport of surface molecules on antigen-presenting cells, and its expression patterns are intimately linked to the functional status of macrophages. Research indicates that CD74-positive macrophages exhibit diminished phagocytic capability and are predisposed to develop into the M2 phenotype (121 ,120). Blocking CD74 can restore the immunosuppressive phenotype and markedly limit the growth of CC cells. The elevated expression levels of IFI30 and TGFBI in macrophages indicate their potential as novel immunological checkpoints (122).

Notably, macrophage subgroups exhibiting elevated HPV16 expression may correlate with favorable prognoses in CC patients. This observation is compounded by the high expression of HPV16 in malignant tumor cells (123, 122). The precise molecular mechanism behind this dual expression pattern and its therapeutic implications require additional investigation.

### 4.3 Dendritic cells

IDO1 is a pivotal immunoregulatory enzyme mostly expressed in immune cells, astrocytes, and some tumor cells, and it significantly contributes to cancer immunoregulation and cellular metabolism. In comparison to precancerous lesions and normal cervical tissues, dendritic cells in CC tissues exhibited a marked elevation of IDO1 and LAMP3 expression (124). This specific expression pattern may contribute to the formation of the immunosuppressive microenvironment in CC. The combination of IDO1 inhibitors and ICIs significantly suppresses IDO1 overexpression and stimulates the proliferation of effector CD8+ T cells, thereby augmenting the efficacy of anti-tumor immunotherapy.

In the examination of dendritic cell-related immune checkpoints, Siglec-10, as a potential immune checkpoint inhibitor, can suppress the function of adaptive T cells via the Galectin-9-mediated signalling pathway, thereby further augmenting the tumor immunological microenvironment of CC (125).

Moreover, plasmacytoid dendritic cells expressing CLEC4C and LILRA4 are pivotal in modulating the immunological response of CC to HPV infection by secreting IFN-α and suppressing viral genome replication during the initial phase of HPV infection (115). During persistent HPV infection, these dendritic cells may assume a pro-oncogenic function by activating NF-κB and MAPK signalling pathways, underscoring their dual role in CC development.

### 4.4 NK cells

NK cells are unique innate immune cells that mediate antiviral and antitumor responses. Blocking immune checkpoints not only saves NK cells from depletion, but also enhances their potent anti-tumor activity (126, 127). Monalizumab is a humanized IgG4 antibody that inhibits NKG2A from binding to its HLA-E ligand, which is overexpressed in tumor cells, and also triggers a natural killer cell-mediated immune response against cancer cells (128). Published findings from phase I clinical study NCT02459301 indicate that monalizumab is well tolerated in individuals with advanced gynecological malignancies, exhibiting minimal therapeutic harm (129).

Furthermore, research indicates that the favorable clinical outcome of CC patients is considerably positively linked with the expression levels of FGFBP2. The increase of FGFBP2 expression can markedly augment the cytotoxicity of NK cells, hence improving the body’s anti-tumor immune response (130). This study elucidates the significant function of FGFBP2 in the TME of CC, while also presenting a novel molecular target for the advancement of NK cell-based immunotherapeutic approaches.

NK cells in the TME acquire CD73 molecules and facilitate immunosuppression through the production of adenosine (131). Recent evaluations have assessed the efficacy of anti-CD73 monoclonal antibodies (oleculumab, NZV930), both as monotherapy and in conjunction with other immunosuppressive agents (e.g. anti-PD-1 and A2AR antagonists), for the treatment of various solid tumors in multiple phase I/II studies (NCT03381274, NCT03454451, and NCT03549000) (126). The impact of anti-CD73 treatment on NK cell functionality requires more investigation.

### 4.5 Mast cells

In the past few years, the regulatory function of MCs in anti-tumor immunity has increasingly garnered interest from the academic community. The study by Zhao et al. demonstrated that elevated ALOX5 expression in MCs is significantly associated with the progression of CC from benign to malignant (132). These MCs may engage in bidirectional contact with CC cells via the TNFRSF12A-mediated signalling pathway. This revelation enhances our comprehension of the mechanism of action of mast cells in the cancer microenvironment and offers a novel research avenue for investigating targeted treatment options centred on mast cell-tumor cell interactions.

## 5 Discussion

By bridging immunological insights with technological innovation, the future of CC treatment lies in precision immunotherapy tailored to the patient’s unique tumor-immune ecosystem. Immunotherapy is slowly becoming one of the most important ways to treat CC. Immunotherapy has come a long way, but there are still a lot of issues that need to be fixed before it can be used to really target and beat immune resistance. CC generally exhibits a relatively low tumor mutational burden, which limits the generation of neoantigens and reduces immunogenicity (133). Furthermore, upregulation of PD-L1, IDO1 expression, and TGF-β signaling can dampen anti-tumor immune responses and limit the efficacy of ICIs (81). We talked about the new immune markers that have been proven by tests *in vitro* and *in vivo*. However, there is still a long way to go before these immune markers can be used in clinical settings.

In summary, the study of immune markers for CC cancer therapy faces many challenges, including little understanding of their interactions in the complex environment of TME and insufficient clinical studies to validate their functionality and potential side effects. We anticipate that with today’s mix of different types of holographic data, such as bulk sequencing, proteomics, spatial transcriptomics, and other technological platforms, to deeply and methodically characterize CC’s immune checkpoints, their functional mechanisms and their biological components will soon become clearer.
